# Sertoli cell-mediated differentiation of male germ cell-like cells from human umbilical cord Wharton’s jelly-derived mesenchymal stem cells in an *in vitro* co-culture system

**DOI:** 10.1186/s40001-014-0080-6

**Published:** 2015-02-03

**Authors:** Lichun Xie, Limin Lin, Qiuliu Tang, Weizhong Li, Tianhua Huang, Xiao Huo, Xiaoshan Liu, Jikai Jiang, Guyu He, Lian Ma

**Affiliations:** Women’s and Children’s Hospital of Shenzhen University, Shenzhen, 518000 China; Department of Pediatrics, Second Affiliated Hospital of Shantou University Medical College, Shantou, 515041 China; Shantou University Medical College, Shantou, 515041 China; Maternal and Child Health Care Center of Pingshan District, Shenzhen, 518000 China; Translational Medicine Center, Second Affiliated Hospital of Shantou University Medical College, Shantou, 515041 China

**Keywords:** Sertoli cells, HUMSCs, Male infertility, Microenvironment, STELLA, VASA, DAZL

## Abstract

**Background:**

Microenvironment signals play a critical role in directing the differentiation of stem cells. Sertoli cells (SCs) provide a unique microenvironment that is essential for germ cell differentiation.

**Methods:**

Our previous study has demonstrated that human umbilical cord Wharton’s jelly-derived mesenchymal stem cells (HUMSCs) could differentiate towards male germ cells *in vitro*, but HUMSC-derived germ-like cells expressed only few germ cell markers. The aim of this study was to investigate the effect of SCs on the differentiation of HUMSCs towards male germ cells using a co-culture system that mimicked the *in vivo* male germ cell microenvironment.

**Results:**

HUMSCs formed clump-like features on SC monolayers after seeding for 3 weeks. Differentiated cells formed round colonies that share the morphological features of spermatogonial colonies. RT-PCR, immunofluorescence, confocal microscopy, and Western blot analyses revealed the expression of early germ cell markers STELLA and VASA and male germ cell-specific marker DAZL in differentiated HUMSCs, confirming the presence of cells with characteristics of male germ cells.

**Conclusion:**

The HUMSC-SC co-culture system mimics a native microenvironment for germ cell colonization without any *in vitro* artificial manipulation and can be used to explore the mechanisms controlling the differentiation of male germ cells from HUMSCs. Male germ cells derived from HUMSCs may be used in the therapy for male infertility.

## Background

Infertility is an emotionally charged issue and male factors are responsible for 40% to 60% of infertility cases [1]. Understanding spermatogenesis is a prerequisite for the elucidation of molecular mechanisms leading to male infertility. Although considerable knowledge has been gained regarding germ cell development in the mouse, and many mechanisms are highly conserved in mammals, there remains a need to investigate human germ cell development directly [2]. Thus, *in vitro* culture systems of human germ cells may open the way to a novel approach to reproductive engineering and eventually novel clinical applications to treat male infertility.

In recent years, the research on derivation of male germ cells from stem cells has opened new perspectives for investigating germ cell development *in vitro*. Several studies have demonstrated that stem cells can be induced to differentiate into cells expressing markers for germ cells [3-5]. However, the progression through the meiotic process is still a challenge in the *in vitro* differentiation of male germ cells from stem cells. The transfection of embryonic stem cell lines with marked or fluorescent proteins allows for characterization of the differentiated germ cells, but the use of transfected lines disqualifies the male germ cells obtained for their application in clinical procedures [6]. The addition of exogenous factors to the culture media such as bone morphogenetic proteins, testosterone and retinoic acid, which play basic roles in germ cell development *in vivo*, seems to help expand the germ cell population and push them to the meiotic process *in vitro*, but it is not enough to direct them through that process properly [6].

Many studies have shown that stem cells differentiate into mature cells based on the signals from the microenvironment [7]. During mammalian spermatogenesis, diploid spermatogonia divide mitotically to provide spermatocytes that proceed through meiosis to haploid spermatids. This process depends on a specific environment provided by the somatic cells of the testis and requires endocrine and auto/paracrine regulation, as well as direct cell–cell interactions [8]. Sertoli cells (SCs) in the mammalian seminiferous epithelium are involved in the regulation of germ cell development by providing nutrients and hormonal signals needed for spermatogenesis [9]. Germ cells are nurtured by SCs, each of which interacts with up to five different classes of germ cells. The SC–germ cell co-culture system has been used to examine the effects of hormones and growth factors on spermatogonial survival and proliferation *in vitro* [10]. Use of a carefully defined SC–gonocyte co-culture system has revealed that germ cell development likely depends on interaction with adjacent SCs [11]. These findings clearly demonstrate that environmental factors are natural inducers of germ cell differentiation. Co-culture of stem cells with SCs may improve the differentiation of mature male germ cells from stem cells.

Human umbilical cord Wharton's jelly-derived mesenchymal stem cells (HUMSCs) are multipotent stem cells with specific mesenchymal characteristics that can be induced to generate different tissues or cells, such as Schwann cells [12], osteogenic cells [13], heart cells [14], skeletal muscle [15], endothelial cells [16], and adipose cells [17]. Unlike mesenchymal stem cells (MSCs) derived from other tissue sources, HUMSCs are more primitive and share some properties unique to fetal-derived MSCs, such as faster proliferation and greater *ex vivo* expansion than adult MSCs [18,19]. Furthermore, HUMSCs can be easily obtained and represent a noncontroversial source of MSCs. In addition, HUMSCs do not express major histocompatibility class II antigens and carry low immunogenicity [20-22]. Therefore, HUMSCs may be an ideal candidate for offering an *in vitro* model to facilitate investigation of germ cell development.

Our previous study has shown that HUMSCs could differentiate towards male germ cells *in vitro*, but HUMSCs-derived germ-like cells only expressed some germ cell markers [23]. Given that SCs provide a specialized microenvironment necessary for germ cell differentiation *in vivo*, we hypothesized that direct attachment between HUMSCs and SCs can improve the differentiation of male germ cells from HUMSCs. In the present study we simulated this environment with a co-culture system composed of HUMSCs and newborn mouse SCs and investigated the effects of co-cultured SCs on the differentiation of HUMSCs into male germ cells *in vitro*.

## Methods

### Animal and human materials

Five- to 7-day-old male Kunming mice were obtained from the Laboratory Animal Center of the Shantou University Medical College (Shantou, China). Animals were maintained in a temperature- and humidity-controlled room and given free access to water and food. Mice were euthanized using CO_2_ inhalation, and testes were collected for subsequent experiments. All animal experiments were conducted in accordance with protocols approved by the Animal Care and Use Committee of Shantou University Medical College.

Human umbilical cords were obtained from women delivering a full-term male infant by cesarean section at the Second Affiliated Hospital of Shantou University Medical College. Signed informed consent was obtained from each mother, and the study was approved by the Human Ethics Committee of the Second Affiliated Hospital of Shantou University Medical College (approval No. SUMC-37-2014).

### Isolation and expansion of human umbilical cord mesenchymal stem cells

HUMSCs were isolated and cultured as previously described [23,24]. The Wharton’s jelly from individual umbilical cords was cut into 1- to 2-mm^2^ pieces, seeded onto 24-well plates (Corning, Midland, Mich. USA), and cultured in DMEM-F12 containing penicillin/streptomycin, 10% fetal bovine serum (FBS; Gibco, USA), 5 ng/mL epidermal growth factor (R & D, USA), and 5 ng/mL basic fibroblast growth factor (Sigma, USA). The cells were left undisturbed for 5 to 7 days at 37°C in a humidified 5% CO_2_ atmosphere, and the medium was replaced every 2 days. When the cells reached 80 to 90% confluence, they were digested with trypsin/EDTA and sub-cultured in culture flasks at a ratio of cells to solution of 1:3.

### Isolation, culture and purification of Sertoli cells

Primary SCs were prepared from decapsulated testes of mice by sequential enzymatic digestion as previously described [25]. The digestion was terminated with DMEM-F12 containing 10% FBS, and the sediment was filtered with a 200-mesh sieve. The cells were aliquoted into 25-cm^2^ culture flasks with medium and incubated at 37°C in a humidified atmosphere of 5% CO_2_. Four hours later, the supernatant containing germ cells was discarded, the dishes were washed thrice with phosphate-buffered saline (PBS), and fresh medium was added. To obtain SC cultures with a purity >95%, cultures were hypotonically treated with 20 mM Tris–HCl (pH 7.4) for 2.5 minutes to lyse any residual germ cells 48 hours after plating. Wells were then washed twice with medium, and cultures were maintained in 5% CO_2_ at 37°C. When adherent cells reached 80 to 90% confluence, they were transferred to new dishes for 3 days and transferred again to eliminate residual germ cells. During culturing, germ cells did not attach to the bottom of culture dishes and could easily be removed by repeated washing. Germ cell–free SC cultures were used for subsequent experiments. The morphological features of SCs were examined by phase contrast microscopy and cells were stained with hematoxylin.

### Preparation of feeder layers

Passage 2 SCs at 80 to 90% confluence were incubated with mitomycin C at 37°C for 2 hours to eliminate mitotic cells and rinsed three times with 5 mL PBS to thoroughly remove mitomycin C. Then trypsin/EDTA solution was added, cells were gently suspended, and the total number of cells was determined by using a hemacytometer. Cell suspensions were plated at 0.5 × 10^5^ cells/cm^2^ in culture dishes and maintained as an adherent monolayer at 37°C in 5% CO_2_. The medium was replaced every 2 days, and cells were cultured for an additional 4 days to form a confluent feeder monolayer with specialized tight junctions.

### Co-culture of human umbilical cord mesenchymal stem cells with Sertoli cells

The HUMSC suspension was seeded on mitomycin-treated SC monolayers to induce germ cell differentiation from HUMSCs. HUMSCs were co-cultured with SCs at a ratio of 1:1. The medium was changed every day. After 1 to 3 weeks, cells were collected for RT-PCR, immunofluorescence and Western blot analyses. Colony diameter and number were determined under an inverted microscope every 7 days during the 3-week culture period.

### RNA isolation and RT-PCR

RT-PCR was performed as previously described [23]. Briefly, total RNA was isolated from HUMSCs and HUMSC–SC co-cultures using Trizol reagent (Invitrogen, New York, USA), according to the manufacturer’s instructions. RT-PCR was performed using the RNA PCR Kit (AMV) 3.0 (TaKaRa, Da lian, China, Japan). PCR conditions were 2 minutes at 94°C, then 35 cycles of 94°C for 30 seconds, 50 to 65°C for 30 seconds, 72°C for 30 seconds, and a final extension for 10 minutes at 72°C. PCR products were resolved by 1.2% (w/v) agarose gel electrophoresis, visualized by ethidium bromide staining, and photographed under ultraviolet light. The human housekeeping gene β-actin was used as a normalization control. The sequences of human-specific primer sets used for PCR were as follows: *VASA* (NM_024415.2, 191 bp), forward 5’-AAG AGG TAG TTT CCG AGG TTG C-3’and reverse 5’-CTT TGT AAC CAC CTC GTT CAC T-3’; *DAZL* (NM_001351, 487 bp), forward 5’-ATC ATC CTC CTC CAC CAC AG-3’ and reverse 5’-GAT TTA AGC ATT GCC CGA CT-3’; *STELLA* (NM_199286, 315 bp), forward 5’-CTC CAC AAA TGC TCA CCG AA-3’ and reverse 5’-GCT CCT TGT TTG TTG GTC TTC T-3’; and β-actin (NM_001101, 396 bp), forward 5’-CAC ACT GTG CCC ATC TAC GA-3’ and reverse 5’-TAC AGG TCT TTG CGG ATG TC-3’.

### Immunofluorescence

For immunofluorescent localization of germ cell markers [23], co-cultured HUMSCs were established on glass coverslips and treated with differentiation or control medium for 7 days. The medium was replaced with fresh medium every 2 days. After 14-day induction, cells were washed thrice with PBS and incubated for 10 minutes in PBS with 1% Triton X-100. Then, cells were blocked for 20 minutes in 5% bovine serum albumin and incubated with human-specific anti-Stella or anti-DAZL antibody (Santa Cruz Biotechnology, Santa Cruz, USA) overnight at 4°C. Cells were then washed in PBS and incubated for 1 hour at room temperature with rabbit anti-goat IgG-TRITC (ZSGB-BIO, Beijing, China). A negative control included cells that were incubated with an antibody of the same isotype as the primary antibody and the secondary antibody. Cells were incubated with DAPI (Sigma) for 5 minutes, washed thrice with PBS, and viewed under a fluorescent microscope and a confocal microscope (laser wavelength: DAPI = 405 nm, Green = 488 nm, Red = 594 nm; Pin hole scale = 50 nm).

### Western blot

Protein was extracted from HUMSCs and HUMSC–SC co-cultures as previously described [26]. The protein concentration of lysates was determined using the BCA Assay Kit (Pierce, Pockford, IL, USA). Protein aliquots were run on 10% SDS-PAGE gels and transferred to nitrocellulose Protran membranes (Whatman, Dassel, Germany). The blots were incubated for 1 hour at room temperature in blocking buffer, incubated with human-specific anti-STELLA, anti-DAZL, or anti-VASA antibody (Santa Cruz Biotechnology) at 1:500 in blocking buffer overnight at 4°C, washed four times in Tris buffered saline solution with Tween-20 (TBST) for 5 minutes each, incubated with secondary antibody (Southern Biotech, Birmington, AL, USA) at 1:10,000 in blocking buffer for 1 hour at room temperature, washed four times in TBST for 5 minutes each, and developed using Super Signal West Pico Chemiluminescent substrate (Pierce) according to the manufacturer’s protocol.

## Results

### Morphology of human umbilical cord mesenchymal stem cells cultured alone

**Figure 1:**
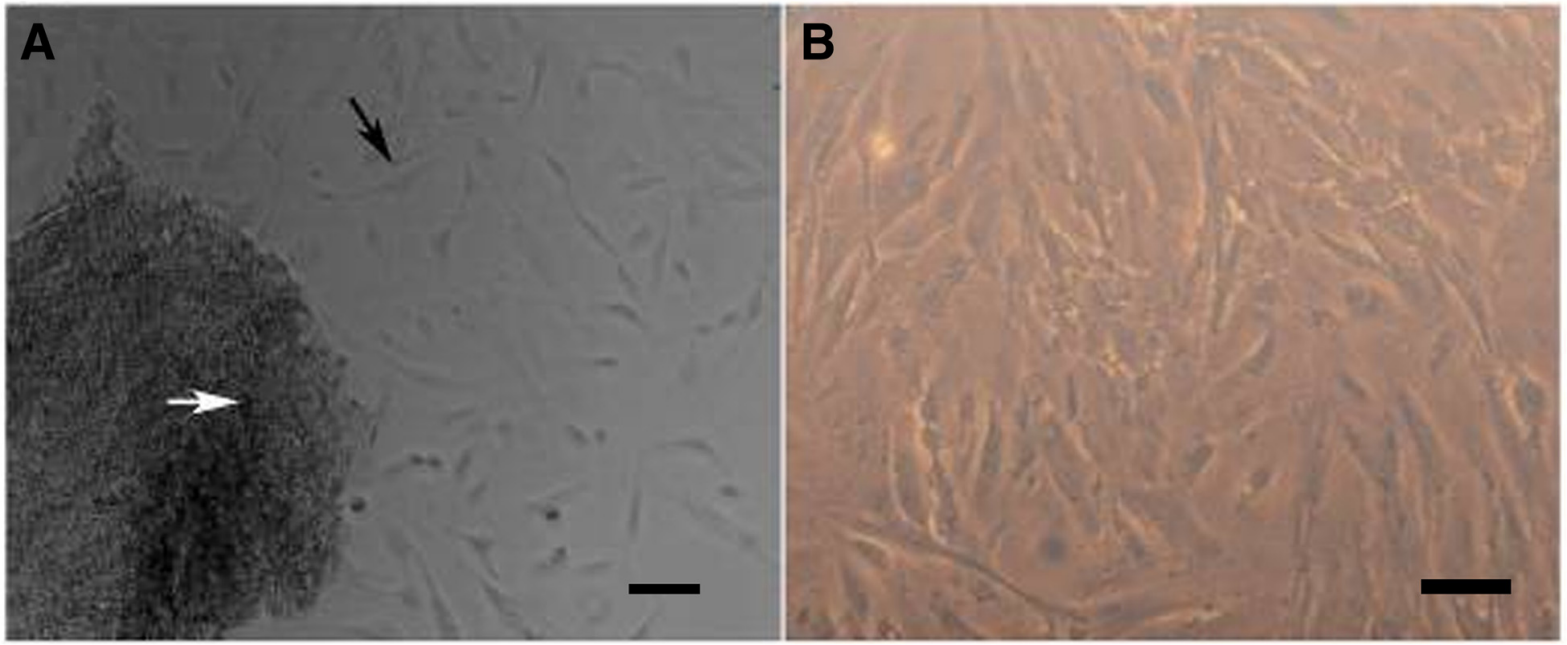
**Morphology of cultured human umbilical cord mesenchymal stem cells (HUMSCs). (A)** Primary HUMSCs on day 7 after culture. HUMSCs (black arrow) migrated out from Wharton’s jelly fragments (white arrow). **(B)** Fibroblast-like HUMSCs at passage 3. (Magnification 100×).

Cultured HUMSCs appeared as spindle-shape cells migrating out from Wharton’s jelly fragments on day 5 to day 7 (Figure 1A). After passage, they appeared as fibroblast-like adherent cells and most were flat, wide and polygonal (Figure 1B). Assessment of the expression of markers associated with endothelial stem cells and adult stem cells by flow cytometry indicated that they possessed the multipotent characteristics of HUMSCs [23].

### Morphology of cultured Sertoli cells

**Figure 2:**
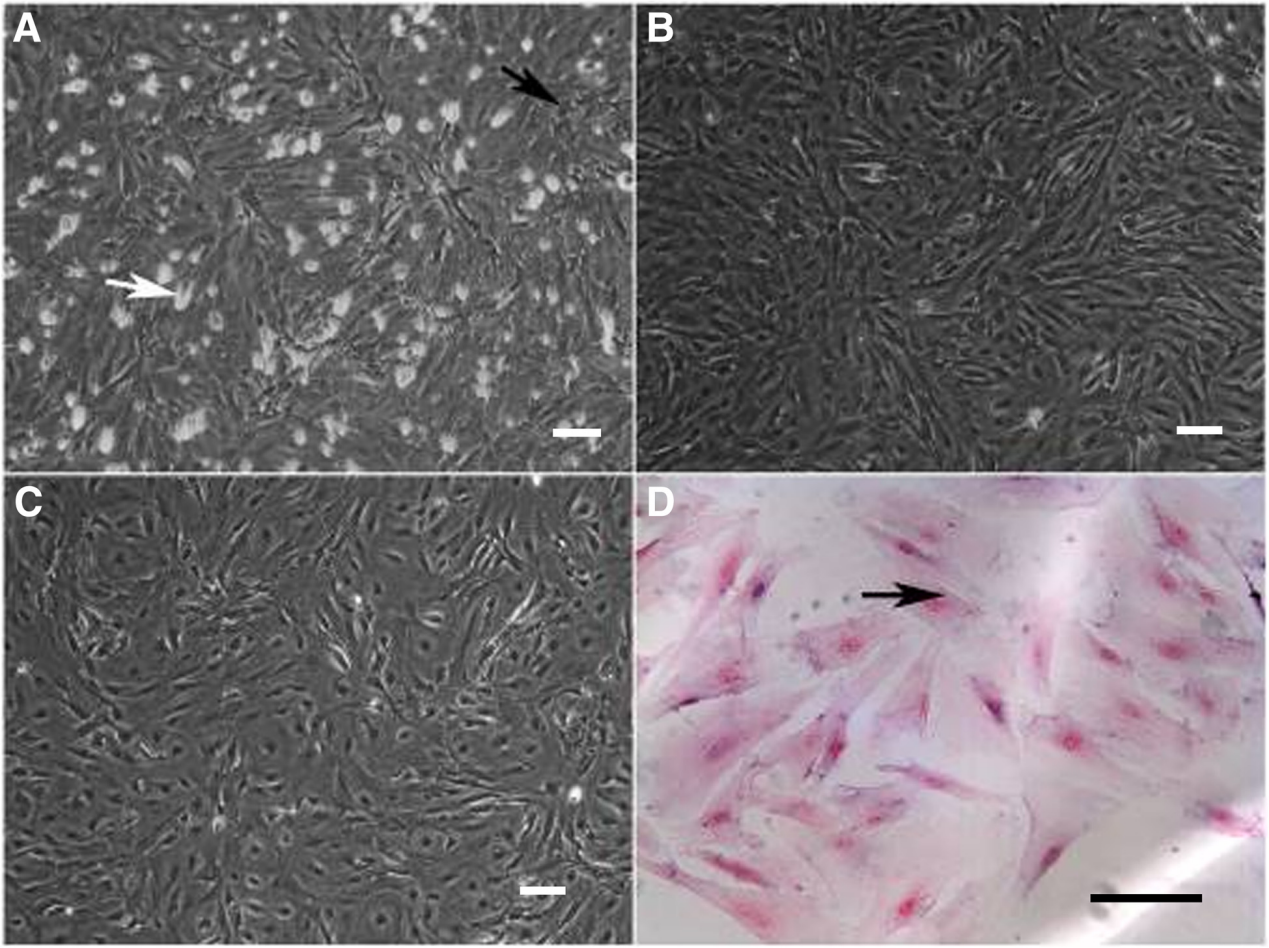
**Morphology of cultured Sertoli cells (SCs). (A)** Primary SCs at 4 hours after plating (magnification 100×). The white arrow indicates a SC, and the black arrow indicates a germ cell. **(B)** Passage 2 SCs with residual germ cells removed (magnification 100×). **(C)** SC monolayer on day 3 after culture (magnification 100×). **(D)** Passage 2 SCs stained with hematoxylin (magnification 200×). The black arrow indicates a SC.

We cultured primary SCs to more than 95% purity by repeated washing, transfer and hypotonic shock. Cell morphology was closely monitored by phase contrast microscopy. Maximal cell adhesion was reached 4 hours after plating (Figure 2A). Passage 2 SCs formed a monolayer on day 3 after plating (Figure 2B). The SCs firmly attached to the bottom of the dish, were irregularly shaped, and had cytoplasmic droplets. These features are in agreement with previously reported features of SCs [27]. After 1 week of culturing, they produced extensions, flattened and attempted to make contact with other cells (Figure 2C). Contamination of SCs by other cell types was also examined. Staining of cells with hematoxylin showed that SCs had larger nuclei than peritubular myoid cells (Figure 2D). The presence of germ cells was not detected after 1 week of culturing.

### Morphology of co-cultured human umbilical cord mesenchymal stem cells

**Figure 3:**
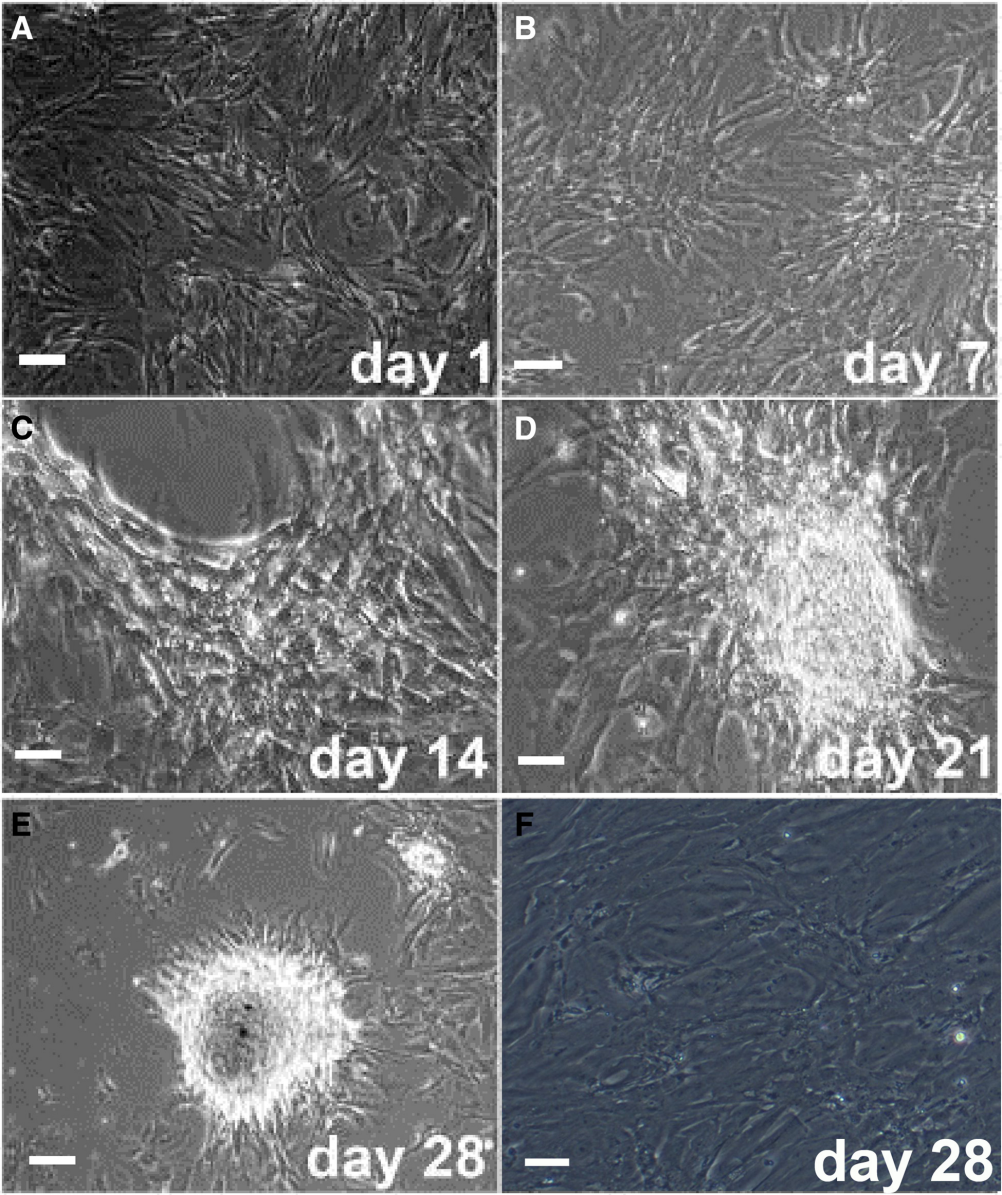
**Morphology of human umbilical cord mesenchymal stem cells (HUMSCs) co-cultured with Sertoli cells (SCs). (A-E**) HUMSC-derived germ cell-like cell colonies formed on a monolayer of SCs at specified time points after co-culture. **(F)** Morphology of SCs on day 28 after culture without HUMSCs. (Magnification 100×).

Isolated HUMSCs were seeded on SC feeder layers. After 24-hour seeding, the transferred cells spread on top of the SC monolayer (Figure 3A). Morphological changes in HUMSCs were observed under a phase-contrast microscope every 3 days. Approximately 1 week later, HUMSCs lost their spindle-like shape, and a few small, loose colonies could be seen (Figure 3B). On day 14, more colonies with germ cell features appeared, and the size of clumped germ cell-like cells seemed larger (Figure 3C). The clumped germ cell-like cells were tightly packed and became flat on day 21 (Figure 3D). On day 28, the colonies showed a typically round shape (Figure 3E). No such changes were observed in untreated SCs or HUMSCs cultured alone (Figure 3F). At passage 5, HUMSCs cultured alone were maintained as spheres.

### Expression of male germ cell-specific markers in human umbilical cord mesenchymal stem cell–Sertoli cell co-cultures

**Figure 4:**
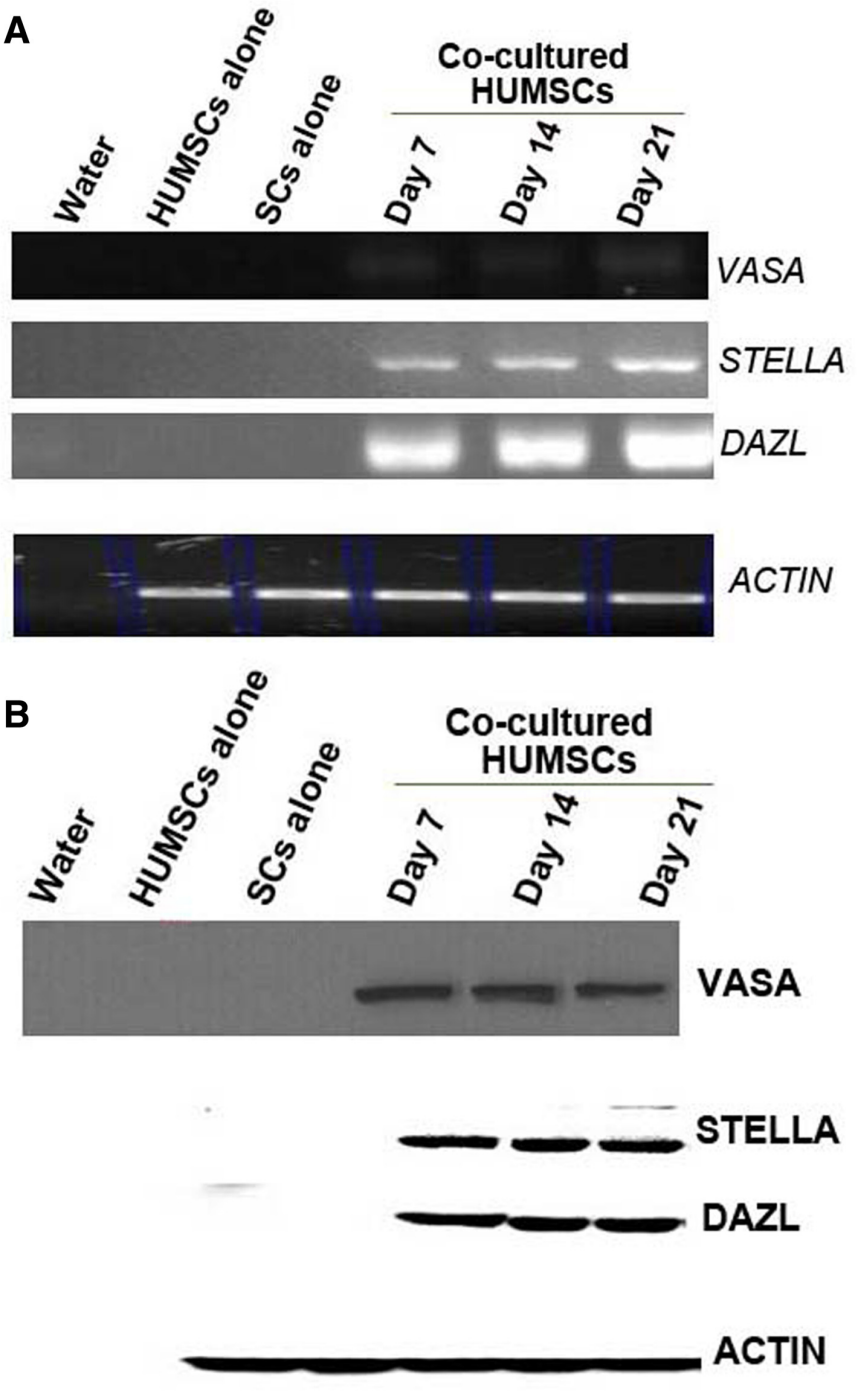
**mRNA and protein expression of germ-cell markers STELLA, VASA and DAZL in human umbilical cord mesenchymal stem cell–Sertoli cell co-cultures.** Total **(A)** RNA and **(B)** protein were prepared from human umbilical cord mesenchymal stem cells (HUMSCs) cultured alone or co-cultured with Sertoli cells (SCs) for different durations and used for RT-PCR and Western blot analyses. Water was used as a negative control, and β-actin (ACTIN) served as a loading control.

Total RNA was isolated from HUMSCs or HUMSC–SC co-cultures on days 7, 14, and 21 to analyze the mRNA expression of germ cell-specific markers. Expression of human-specific *STELLA*, *VASA*, and *DAZL* mRNAs could be detected at various time points in co-cultured HUMSCs, but not in HUMSCs cultured alone (Figure 4A).

To examine whether the protein expression of STELLA, DAZL and VASA in HUMSC-derived clumps of male germ-like cells was consistent with their mRNA expression at different time points, Western blot was performed. As shown in Figure 4B, the protein expression patterns of these germ cell-specific markers were similar to their mRNA patterns.

**Figure 5:**
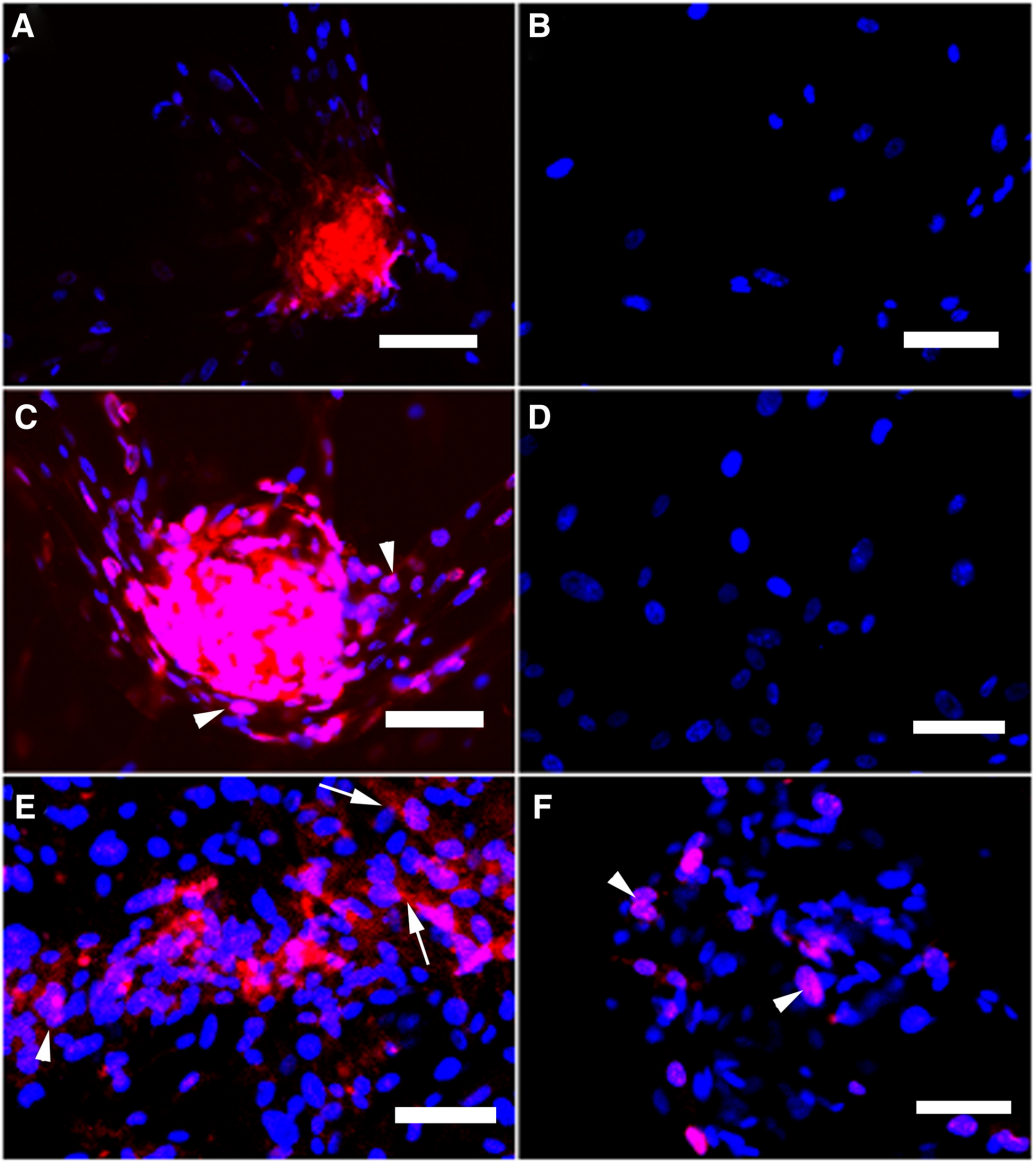
**Immunofluorescence localization of STELLA and DAZL in human umbilical cord mesenchymal stem cell (HUMSC)-derived germ cell-like cells.** Staining for STELLA (red) **(A,** **B,** **E)** and DAZL (red) **(C, D, F)** in differentiated cells **(A, C, E, F)** on day 14 after co-culture and HUMSCs cultured alone **(B, D)** were performed and examined by immunofluorescence microscopy **(A-D)** and confocal microscopy **(E, F)**. Nuclei were counterstained with DAPI (blue). Arrowheads indicate nuclear staining, and arrows indicate cytoplasmic staining. (Magnification 200×).

We next examined the expression and localization of human-specific STELLA and DAZL proteins in clump-forming male germ cell-like cells after 14-day culture by immunofluorescence and confocal microscopy (Figure 5). We chose this time point to assess protein expression because the mRNAs for both germ cell markers were expressed at this stage. Both markers were detectable in co-cultured HUMSCs, but not in HUMSCs cultured alone (Figure 5B, D). Expression of human-specific STELLA in co-cultured HUMSCs was found to be primarily in cytoplasmic and nuclear regions, while the DAZL protein was localized to the nucleus.

## Discussion

SCs provide a microenvironment that is essential for germ cell proliferation and differentiation. In the present study we created an environment *in vitro* that mimicked the *in vivo* male germ cell microenvironment to help HUMSCs differentiate into male germ-like cells using a co-culture system. Morphological, immunofluorescence, and molecular data showed the differentiation of male germ cell-like cells from HUMSCs. Differentiated clumps of germ cell-like cells expressed human early germ cell markers STELLA and VASA and male germ cell-specific marker DAZL. Strikingly, DAZL was not expressed and VASA was expressed late when HUMSCs were cultured alone in conditioned media [23]. This finding suggests that mechanical signals produced through direct cell-to-cell contact between SCs and HUMSCs in the co-cultured scenario might be critical in the differentiation process.

The processes that control HUMSC differentiation are complex, and the signals involved in these processes are largely unknown. However, it is known that the microenvironment of HUMSCs is critical for their differentiation. Through the use of co-culture techniques, numerous microenvironment factors involved in cellular differentiation have been identified and broadly characterized as either chemical or physical. Chemical signals include protease, cytokines, hormones and other factors that are produced by the cells in the microenvironment [28]. In addition, microvesicle/exosome-mediated transfer of transcription factors and nucleic acids between cells may be involved in directed differentiation [29,30]. Particularly, SCs secrete a wide range of growth factors, cytokines and other soluble elements that are elaborated for spermatogenesis [31]. In our study, HUMSCs and SCs were co-cultured at a ratio of 1:1 and SCs were first treated with mitomycin C to restrain their proliferation and keep the function of secretory factors.

In addition to chemical molecules, direct cell-to-cell contact also plays an important role in controlling the differentiation of male germ cells from HUMSCs. Mechanical signals are complex and may include stimulation of receptors through direct contact with neighboring cells or by the components of the extracellular matrix, tight junctions, desmosomes, gap junctions and the influences of cell stretch or other forces and perhaps even other local signals [28]. SCs are polarized epithelial cells that interact with one another via a unique, large complex of basolaterally located junctions. This complex consists of various junction types, including adherent and desmosome-like junctions, and is characterized by testis-specific SC structures termed “ectoplasmic specialisations” [28]. In the present study, we found that HUMSCs lost their spindle-like shape and formed colonies after they were co-cultured with SCs for a period of time. These changes might result from mechanical stretch imposed by neighboring cells, or some protein factors secreted by SCs may serve as signals for communication between HUMSCs and SCs in the co-culture system. Connexins expressed by SCs might activate the dormant channels that open with cell-to-cell contact and aid in the transfer of intracellular signaling molecules, which may in turn activate the expression of differentiation-associated genes and alter cell phenotypes [28]. We believe that direct cell-to-cell contact between HUMSCs and SCs plays a critical role in the differentiation of male germ cells from HUMSCs.

During co-culture, differentiating cells formed round colonies which share the morphological features of previously described spermatogonial colonies [32]. The specific mRNA and protein expression of male germ cell markers STELLA, VASA and DAZL in HUMSC-derived clumps confirms the presence of cells with characteristics of male germ cells, as was previously reported [27,32-35]. STELLA has been considered a potential marker for embryonic stem cell-derived germ cells or may specifically mark cells committed to the germ-cell line [36]. It is involved in initiating germ cell competence and specification and in the demarcation of primordial germ cells from their somatic neighbors [34]. *VASA* (Mvh/DDX4) encodes an ATP-dependent RNA helicase, which is specifically expressed in differentiating germ cells from the late migration to postmeiotic stage, with the gene being specifically expressed in early (PGCs) (primordial germ cell). Loss of VASA function causes deficient proliferation and differentiation of male germ cells [34].

We also showed the nuclear expression of DAZL in differentiating HUMSCs. DAZL, present in spermatogonial nuclei [34], is a specific molecular marker for spermatogonia and is essential for PGC development, because DAZL-knockout mice lack a germ-cell population [36]. DAZL protein is a germ cell-specific RNA-binding protein essential for gametogenesis. In humans, loss of Y-chromosomal *DAZL* genes results in oligozoospermia or azoospermia. The *DAZL* genes are strong candidates for azoospermia factor, one of the most common genetic causes of male infertility [3]. One of the protein products of the *DAZL* gene is expressed throughout most of the life of germ cells and is required for the development of PGCs and for the differentiation and maturation of germ cells from PGCs onward.

This study had several limitations. First, we did not characterize specific types of germ cells differentiated from HUMSCs, such as Oct4-expressing cells. Second, only a few germ cell markers were detected in this study. Finally, given the time-dependent expression of male germ cell markers, meiotic and post-meiotic male germ cell markers might express if a longer co-culture was performed. Future studies are needed to carefully address these issues.

## Conclusions

In conclusion, herein we have developed a simple and efficient cell co-culture system to induce germ cell differentiation from HUMSCs. This co-culture system mimics a native microenvironment for germ cell colonization without any artificial manipulation *in vitro* and can be used to explore mechanisms controlling the differentiation of male germ cells from HUMSCs. Male germ cells derived from HUMSCs may be used in therapy for male infertility.

## Abbreviations

DMEM-F12: Dulbecco’s modified eagle’s medium/Ham's nutrient mixture F-12
FBS: fetal bovine serum
HUMSC: human umbilical cord Wharton’s jelly-derived mesenchymal stem cell
MSC: mesenchymal stem cell
PBS: phosphate-buffered saline
PGC: primordial germ cell
RT-PCR: reverse transcription-polymerase chain reaction
SC: Sertoli cell
TBST: Tris buffered saline solution with Tween-20
